# Gold Nanoparticles Synthesis and Antimicrobial Effect on Fibrous Materials

**DOI:** 10.3390/nano11051067

**Published:** 2021-04-21

**Authors:** Behnaz Mehravani, Ana Isabel Ribeiro, Andrea Zille

**Affiliations:** 2C2T-Centro de Ciência e Tecnologia Têxtil, Campus de Azúrem, Universidade do Minho, 4800-058 Guimaraes, Portugal; behnaz.mehravani@yahoo.com (B.M.); afr@2c2t.uminho.pt (A.I.R.)

**Keywords:** gold nanoparticles, in situ, textiles, antibacterial properties, functionalization

## Abstract

Depositing nanoparticles in textiles have been a promising strategy to achieve multifunctional materials. Particularly, antimicrobial properties are highly valuable due to the emergence of new pathogens and the spread of existing ones. Several methods have been used to functionalize textile materials with gold nanoparticles (AuNPs). Therefore, this review highlighted the most used methods for AuNPs preparation and the current studies on the topic in order to obtain AuNPs with suitable properties for antimicrobial applications and minimize the environmental concerns in their production. Reporting the detailed information on the functionalization of fabrics, yarns, and fibers with AuNPs by different methods to improve the antimicrobial properties was the central objective. The studies combining AuNPs and textile materials have opened valuable opportunities to develop antimicrobial materials for health and hygiene products, as infection control and barrier material, with improved properties. Future studies are needed to amplify the antimicrobial effect of AuNPs onto textiles and minimize the concerns related to the synthesis.

## 1. Introduction

Metal nanoparticles have demonstrated unique physical and chemical properties unlike those in their bulk state. This is attributed to the quantum size effect resulting in specific electronic structures [1,2]. For antimicrobial purposes, silver nanoparticles (AgNPs) have presented particular interest. However, in vitro studies have demonstrated their toxic effects on liver, neuronal, epithelial, and murine stem cells. AgNPs have also shown toxicity in aquatic organisms and accumulation in plants, which allow their introduction into the food chain. These facts triggered the research for other metals to obtain antimicrobial effects [3]. Gold nanoparticles (AuNPs) emerged as an alternative due to their higher biocompatibility and facility of surface functionalization [4]. Additionally, AuNPs present intense plasmon resonance and suitable electrical, magnetic, and thermal conductivity and chemical stability (either in atmospheric conditions or living tissues, being resistant to oxidation) [5]. In pharmacology, they present attractive anti-HIV, anti-angiogenesis, anti-malarial, anti-arthritic, and some antimicrobial activity. Biomedical applications include drug delivery, gene therapy, catalysts for medical therapy, and diagnostics [6,7]. Regarding the antimicrobial properties of the AuNPs, studies have demonstrated that several parameters condition its activity [8]. The abovementioned properties/activities of AuNPs are dependent on their physicochemical characteristics provided by the surface composition, size, and shape. During the AuNPs synthesis, variables such as the reaction temperature, stirring rate, the ratio of the gold to a reducing agent, and the use of stabilizing agents and surfactants influences the final characteristics of nanoparticles [9]. Specifically, the AuNPs surface, where the external atoms are bonded to the internal atoms, opens opportunities for interaction with donor–acceptor species or ligands, promoting variable surface charges. In addition, the use of low concentrations of surfactants is beneficial to obtain the desired morphologies and avoid agglomeration [9,10,11]. Another common approach is the AuNPs functionalization with molecules attached to their surface by chemisorption, electrostatic attraction, hydrophobic interaction, or chemical bonds. The head group of the ligands are able to improve the interaction of AuNPs and the external environment to improve the desired effects [12]. Thus, synthesizing AuNPs with specific characteristics is highly valued.

Synthesis of gold nanoparticles dates to 1951 where Turkevich used sodium citrate as reducing agent for producing gold nanoparticles. Since then, researchers have used other reducing agents such as gallic acid, hydrogen peroxide, hydrazine etc. Later, a two-phase method was proposed by Brust-Schiffrin in 1994. However, usage of citric acid, sodium borohydride (NaBH_4_), polyethylene glycol (PEG), hexadecyltrimethylammonium bromide (CTAB), trioctyl-phosphine (TOPO), and oleyl amine (OAm) showed to be toxic, harmful, irritating, flammable, or hazardous to the environment. Therefore, green synthesis methods were introduced recently, where chemical reducing agents are being replaced by plant extracts, bacteria, yeasts, fungi, and enzymes [13,14]. 

Over the last years, the functionalization of textile materials with nanoparticles became more and more important in several applications due to the demand for added value and durable products. Nanotechnology can provide easy-care, stain repellence, electrical conductivity, and static elimination to fibers without compromising their comfort and flexibility. The use of nanoparticles is a multidisciplinary approach to provide UV blocking, antimicrobial, water repellent, colorant, flame retardant, sensing, and self-cleaning properties to textiles [15]. Particularly, the antimicrobial textiles with improved functionalities find several applications, namely, in health and hygiene products, infection control, and barrier material [16]. 

Various techniques have been used by researchers to functionalize natural and synthetic fibers with AuNPs such as sputtering, electrostatic assembly, chemical reduction in solution, dip-coating, electroless plating, drop and dry, biosynthesis, and print pasting method. These methods can be categorized into two ways, the first path includes already prepared nanoparticles, and the second path comprises in situ reduction carried out in the existence of fibers. In situ reduction methods was more often used as it can be done in single step, where a metal precursor solution is reduced with the help of reducing agents such as sodium citrate or NaBH_4_ in the presence of fibers. Several natural fibers such as cotton, linen, and silk or synthetic fibers such as polyamide 6, polyethylene terephthalate, polybutylene succinate, and polyvinylidene fluoride were used to produce the Au-textile nanocomposites using in situ chemical reduction. The other simple and cost-effective methodology are dip-coating and electrostatic assembly. In these methods, for a certain duration of period, the textile fibers are immersed in a AuNPs solution and the nanoparticles are settled on the fibers. The solution can be vigorously stirred maintaining certain temperature for getting effectively coated on the surface of the fibers. For homogenous coating, pre-treatment of fibers with processes such as electrostatic assembly is worth, where it will avoid the electrostatic repulsion between the colloidal particles and textile fibers. The seeded growth method is another approach where Au nano seeds are generated using reducing agents in the presence of textile fibers and Au/textile fiber seeds are used as the aqueous solution to grow the Au nanoparticles [17]. 

Accordingly, this review provides an overview of the most used methods for AuNPs synthesis and the current reports. Two main concerns exist in AuNPs synthesis, one related to the development of AuNPs with appropriate physicochemical properties to provide antimicrobial action and another related to the environmental impact of AuNPs production. Next, the main focus of this work was to provide a comprehensive insight into the strategies to functionalize textile materials with AuNPs, including the studies with and without antimicrobial purposes. 

## 2. Research Methods

The present review was written based on other review articles, research articles, book chapters, and conference proceedings, encompassing AuNPs and textile materials. The different keywords are chosen for searching the articles such as “gold nanoparticles”, “antimicrobial properties of gold nanoparticles” and “synthesis of gold nanoparticles” and “deposition of gold nanoparticles” in the Scopus database for the last 10 years from 2010 to 2020. For this, 14331, 7921, 3667 articles are extracted for the keyword “gold nanoparticles” for the year 2020, 2015, and 2010, respectively. These numbers suggested that there is a lot of research activity happening on the topic of gold nanoparticles and there is a rapid growth in number of articles published from 2015. Searching the keyword “antimicrobial properties of gold nanoparticles,” it retrieved 2312, 702, and 170 articles for the year 2020, 2015, and 2010, respectively. The rate of applications was improved from 2015 and researchers focused on functionalizing the textiles using AuNPs since then to improve the antibacterial properties of textiles. The keyword “deposition of gold nanoparticles” has retrieved 5142, 2882, and 1501 articles for the years 2020, 2015, and 2010, respectively. There is good growth in the number of articles published from 2015 onwards, and it suggests that gold nanoparticles are coated onto textile for improving their functionalities to use them in various applications. The irrelevant studies were ignored, and the rest of the articles was chosen to write this review focusing on presenting the information about the different methods for the synthesis of gold nanoparticles such as in situ synthesis and ex situ synthesis. Besides, this article investigated and reviewed the different methods to coat the gold nanoparticles onto the textiles, fabrics, yarns, fibers, nanofibers, and membranes.

## 3. AuNPs Synthesis

As mentioned above, the antimicrobial activity of AuNPs is dependent on their size, shape, surface composition, and charge. The synthesis of AuNPs with specific characteristics is of great technological interest [11,18]. Several variations on traditional methods have been proposed to control the properties of AuNPs by incorporating different reactants, stabilizing agents, or ligands and by changing reaction conditions including temperature, pH, concentration of reagents, and solvents [19]. In this section, a brief presentation of the different methods used for AuNPs is carried out, centering on those applied in AuNPs synthesis for in situ or ex situ deposition on textile materials.

The methods described in the literature for the synthesis of AuNPs are mainly categorized into two approaches, i.e., top-down and bottom-up approaches. The significant difference between these two methods was the starting material of the nanoparticle preparation. In a top-down approach, bulk material was used as starting material and the particle size was reduced to nanoparticles by different physical, chemical, and mechanical processes. In the bottom-up approach, atoms or molecules were used as the starting materials [20]. In both, several physical or chemical treatments were used to convert the bulk material into small nano-sized particles [21]. Top-down methods are easy to accomplish but they are not suitable for producing defined shaped and very small size particles, and the major issue with this approach was the change in surface chemistry and physicochemical properties of nanoparticles [22]. In bottom-up methods, nanoparticles are formed from atoms, molecules, or small particles. Nanostructured building blocks are formed initially and then they are assembled for producing the final nanoparticle [23]. 

**Figure 1:**
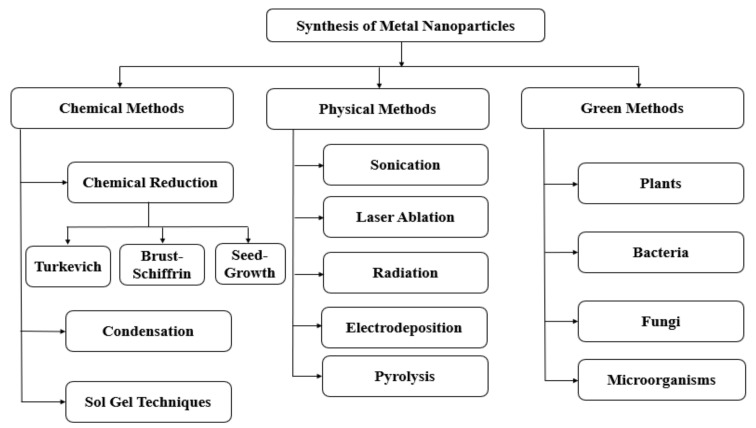
Different methodologies for synthesis of nanoparticles (adapted from [26]).

The methods can also be divided into physical, chemical, and biological (Figure 1). Sonication, laser ablation and radiation come in the category of physical methods, whereas condensation, sol–gel method, and reduction (electrochemical, chemical, or photochemical) come under chemical methods. Nevertheless, firm guidelines act as barrier to use old methodologies that involve the usage of toxic chemicals in the synthesis of AuNPs. Hence, there is a shift in the researchers to incline towards green synthesis of nanoparticles named “biological methods” where they use agents from nature [24,25]. 

Reduction methods in aqueous medium tend to form quasi-sphere AuNPs. This morphology has the smallest surface area compared to others, making the reduction the most used method to obtain AuNPs [27]. Two steps are involved in the reduction method, nucleation, and successive growth. The process is described as in situ synthesis when the nucleation and successive growth are completed in one step. If both steps occur in two different steps, then it is termed as seed-growth method. In situ synthesis is preferred for the synthesis of spherical or quasi-spherical AuNPs and the seed-growth method is preferred for producing various sizes and shapes [1]. The preparation of AuNPs by conventional chemical reduction consists of two vital parts. First, the reduction step is performed using agents such as borohydrides, aminoboranes, hydrazine, formaldehyde, hydroxylamine, saturated and unsaturated alcohols, citric and oxalic acids, polyols, sugars, hydrogen peroxide, sulfites, carbon monoxide, hydrogen, acetylene, and monoelectronic reducing agents including electron-rich transition-metal sandwich complexes. The stabilization in the second step is provided by agents such as trisodium citrate dihydrate, sulfur ligands, phosphorous ligands, nitrogen-based ligands, oxygen-based ligands, dendrimers, polymers, and surfactant (in particular, cetyltrimethylammonium bromide-abbreviated CTAB) [28]. However, chemical methods frequently generate toxic by-products that compromise living organisms and the environment. Hence, new green biological methods have emerged during the last years. Biosynthesis focuses on environmentally friendly methods that reduce or eliminate the use of hazardous toxic substances, providing biocompatible AuNPs [29]. AuNPs provided from biological methods may use fungi, bacteria, or plants as reducing agents [30]. Biological methods also have some disadvantages, namely, high polydisperse, reaction parameters difficult to control and reproduce (pH, reaction time, and concentration) and safety issues when fungi or bacteria are used [31]. Despite the need for optimization of biological methods, the researchers are focusing on these methods to avoid risky and costly processes to synthesize AuNPs [32]. Additionally, more studies are needed to review the antimicrobial properties of different synthesized AuNPs and clarify the different mechanism of actions.

## 4. Methods to Prepare Textile Materials Functionalized with AuNPs

**Figure 2:**
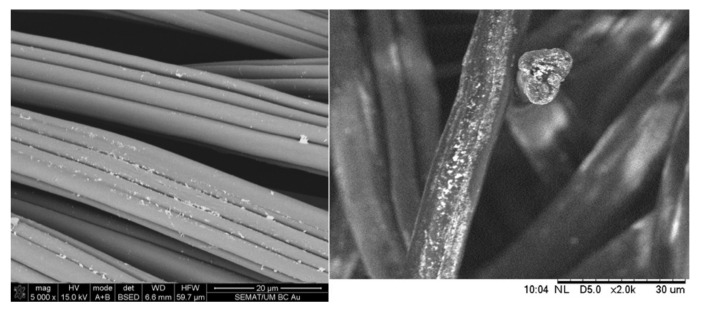
Gold nanoparticles (AuNPs) deposited onto synthetic fibers.

Textile industry has benefited from the development of new concepts of applications, particularly by their conjugation with nanomaterials. Textile materials are included in several advanced and smart applications encompassing woven, knitted, and non-woven fabrics; fibers; yarns; threads; nanofibers; scaffolds; and membranes. The incorporation of AuNPs is interesting to obtain fabrics with innovative coloration, filtration, antimicrobial, conductive, UV protection, sensory, and catalytic properties as presented in this section (Figure 2). Driven by these excellent properties, during the last years, several scientific publications have reported methods to perform this combination.

**Figure 3:**
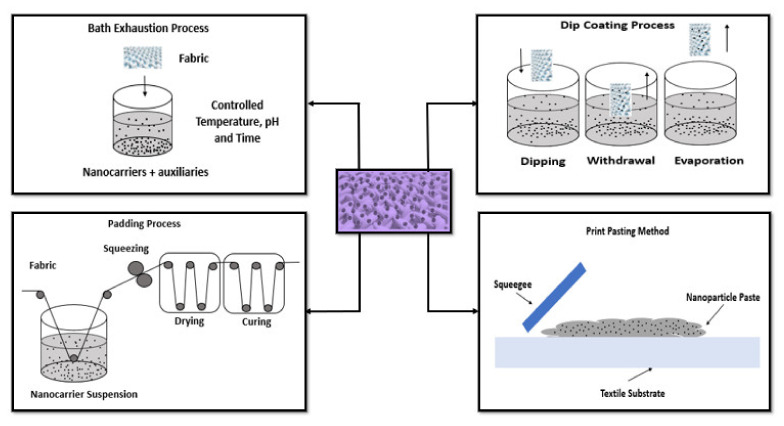
Some methods to functionalize textile materials with AuNPs (adapted from [33,34,35]).

The functionalization of textile surfaces with AuNPs have been performed by several methods including exhaustion, padding, dip coating, electroless, screen printing, dropwise, immersion, sonication, and electrospinning (Figure 3). 

Exhaustion is one of the older process, in which the textile material is placed in the nanomaterial’s solution and the nanomaterials are adsorbed onto the surface of the fabric. Parameters such as exhaustion temperature, time, pH, concentration of nanomaterial solution, and addition of auxiliary agents play a crucial role in this process. Once the fabric is taken out from the solution, it is washed and dried [33,36]. The padding process is a traditional method and involves the use of a pair of rollers where the textile is immersed in a nanomaterial dispersion and then it is directed to move in between the rollers. This facilitates the penetration of nanomaterial into the textile and removes the excess of the solution from the textile. The pressure of the rollers and the speed of the fabric moves through the roller are vital in this procedure. Once the textile is passed out of the roller, it is then dried and cured, which helps to keep the nanomaterial on to the surface [33,37,38]. Dip coating is another impregnation method to coat nanoparticles on fibers or fabrics. The materials are dipped into the coating bath. The fibers/fabrics obtained are dried and may be cured, which enhance the adhesion between nanomaterial and the fibers/fabrics [33,39]. Electroless deposition is a common method used to deposit metal on nonconductive surfaces. It is based on surface charge activation and covers the layer with a catalyst. This process includes reduction of metallic ions to pure metal in the presence of a catalyst on to a surface. The electroless deposition will be carried out, as long as it is immersed in the bath. It is a simple technique and facilitates the adhesion of the nanoparticles onto the surface [33,40,41]. The print method was used by very few authors using a AuNPs paste made up of thickener, binder, and nanomaterial. The mixture can be applied via flat-to-flat method using a squeegee or screen printing on a curved surface using a rotating cylinder [33,42]. In the drop-wise deposition, the AuNPs are dispersed in a volatile liquid that is deposited by drop-on-demand ink jet heads. After that, the materials pass through a heating and evaporation process. In this final step, the particles deposition is promoted [43]. Immersion is a simple technique based on the direct dipping of the material into nanoparticles solution and subsequent diffusion. This process is time dependent and may require several minutes. The success of the deposition depends on the affinity between the materials surface and nanoparticles [44]. Sonication deposition is based on ability to generate acoustic cavities that undergo implosive collapse, discharging enormous energy in the form of high temperature and pressure. These facts produce shock waves, microjets, turbulence and shear forces that enhance the diffusion of nanoparticles and increase the kinetic energy of the system to overcome their ionic repulsion forces. Thereby, it reduces the deposition time and increases the surface coverage of nanoparticles [45]. Lastly, electrospinning also can be used to introduce nanoparticles into textile materials by mixing them with the polymer solution before the fiber formation. The polymer solution is charged and ejected through a spinneret under a high-voltage electric field that solidifies or coagulates to form filaments [46,47]. 

Herein, the studies and methods evolving AuNPs and textile materials are underlined (Table 1). The studies were grouped first depending on the textile materials type and second depending on the method to synthesize the AuNPs. The main aim was to emphasize the studies that focus on textiles functionalized with AuNPs to understand the developed techniques according to this purpose to promote novel studies. Despite some of these studies are not connected with the development of antimicrobial textiles, it will be possible to comprehend other approaches, applied in other fields, to open new possibilities in the development of antimicrobial textiles materials. Thus, the most important methods and cost-competitive studies were considered.

### 4.1. Functionalization of Fabrics with AuNPs

#### 4.1.1. Chemical Reduction Method without Pre-Treatments on Fabrics

Within the methods to produce AuNPs to functionalize textiles, chemical reduction is one of the most applied. This method is preferred due to its simplicity, but the current trend is to replace it with more environmental-friendly and inexpensive methods. Researchers often use the traditional two-step functionalization (synthesis and posterior deposition) but also in situ methodologies, where the synthesis and deposition of gold nanoparticles on fabrics takes place in single step. The common reducing agents used in the two-step process are NaBH_4_ and sodium citrate. When the two-step method is applied, different methodologies were reported for deposition such as drop-wise deposition, exhaustion, padded, impregnation, and printing of the fabrics with AuNPs dispersion. Kam Ling Chan et al. (2016) have synthesized AuNPs using chloroauric acid (HAuCl_4_) as precursor salt, NaBH_4_ as reducing agent, and sodium citrate as a capping agent. They were coated on fabrics made with cotton, silk, and wool using drop-wise deposition [48]. Coating of keratin protein in combination with gold nanoparticles onto the cotton fabric by padded method was attempted by O Shanmugasundaram et al. (2018) to improve the antibacterial property. Porosity and water absorbency of fabric was decreased with the coating of keratin and AuNPs. AuNPs were synthesized using a chemical reduction method where HAuCl_4_ and trisodium citrate were used as precursor salt and reducing agent, respectively. The synthesized nanoparticles were measured and found to be in the size of 8–30 nm with mean size as 14 nm [49]. T. Abou Elmaaty et al. (2018) have used a simple method, printing and paste, to coat the AuNPs on to the polyester and cotton fabrics. The gold nanoparticles were synthesized using gold (III) chloride hydrate and sodium citrate. Thereafter, the solution was made into paste, and it was printed using flat screen technique. TEM observations revealed that the average diameter was observed in the range between 13 and 20 nm [42]. Yidan Zheng et al. (2013) have produced silk and cotton fabrics coated with gold nanorods for coloring. Au nanorods with the size of 19 nm were synthesized using seed-mediated growth method where they have used HAuCl_4_, NaBH_4_, and CTAB as precursor solution, reducing agent, and stabilizing agent, respectively. The Au nanorods were deposited onto the fabrics by immersing them in the Au nanorod solutions [50].

The in situ approach allowed to remove one step to functionalize the fabrics. Here, chemical reducing agents are added to the gold salt solution or the chemical groups on the fiber surface may act as reducing agent itself. Xia Lin et al. (2017) have produced AuNPs via reduction method using HAuCl_4_ as a precursor solution and trisodium citrate as a reducing agent, where pH value was maintained at 5.0–6.5. The nylon fabrics was functionalized with gold nanoparticles by immersion and heating process. It was observed that the color of the fabric became lighter with an increase in the concentration of the reducing agent [51]. Chitosan being nontoxic and cationic in nature, Iris O. Silva et al. (2019) treated knitted fabric to improve the binding effect of nanoparticles. The AuNPs were chemically synthesized, and NPs with spherical shape and approximately 35 nm in size were obtained. Then, the team immobilized the AuNPs onto the soybean knitted fabric treated with chitosan by exhaustion method. In this process, HAuCl_4_, sodium citrate dihydrate, and chitosan acted as precursor salt, reducing agent, and stabilizing agents, respectively. The fabrics developed showed improved thermal stability [52]. Liheng Gao et al. (2020) have produced single-layered Ag-Au-Pt nanocrystal ternary coated biomass textiles using polymer-driven self-assembly strategy. In order to improve the water solubility, oxidative resistance, and affinity to biomass, metal nanoparticles, are transformed into homogenous hyperbranched poly(amide-amine) (HBPAA) encapsulated nanoparticles. In this study, the AuNPs were produced by in situ synthesis, where HAuCl_4_.4H_2_O and NaBH_4_ were used as precursor salt and reducing agent, respectively. AuNPs with a diameter of 6.64 nm were mixed with other nanoparticles, and the textiles were impregnated in the solution [53]. Hanan B. Ahmed et al. (2017) produced AuNPs and coated a silk fabric by immersion with constant shaking and temperature to enhance NPs adhesion and, consequently, the antibacterial properties. In the process of synthesizing AuNPs by in situ reaction, AuCl_3_ was used as a precursor salt, silk macro molecular chains were used as reducing agent, and silk fabric itself acted as stabilizing agent. Hydrogen peroxide was used to activate the silk macro molecules, and the size of the AuNPs was in the range of 22–66 and 18–49 nm [54]. Ultrasonic treatment was used by Lin Zhou et al. (2019) for the better deposition of gold nanoparticles on to the silk fabric. HAuCl_4_ and sericin in silk were used as precursor salt and reducing agent, respectively, to produce AuNPs. The AuNPs were deposited on the fabric by soaking the fabric in the solution and using ultrasonic treatment. The AuNPs found to have spherical and ellipsoidal shape in the size of 11 ± 4 nm [55]. A few works in the literature exist with this last method, which can be a promising approach to reduce the chemicals during the NPs synthesis, the preparation time, and the energy required. More studies are needed to provide toxicological tests and adapt to industrial processes.

#### 4.1.2. Chemical Reduction Method with Pre-Treatments on Fabrics

Pre-treatments of fabrics are vital to enhance the reactivity onto their surface and enhance the AuNPs deposition rate or to act in synergy with AuNPs improving a specific property. Two different studies emerged in the literature, the first one was the fabric surface activation using plasma treatments and the other one was the pre-functionalization with minerals, by silanization with alkoxysilane molecules or by cross-linking agents. It was reported by Nina Radic et al. (2012) that dielectric barrier discharge and diffuse coplanar surface barrier discharge was used to enhance the deposition of AuNPs. The AuNPs were synthesized by the reduction of HAuCl_4_ solution by gallic acid without stabilizer and coating onto the fabric by immersion of the fabric in the solution containing AuNPs. The size of the particles deposited onto the fabrics was in the range of 30–60 nm, and it was observed that they were smaller in solution, which indicates some agglomeration on the fabric [56]. Another methodology was reported by Makoto Ikegami et al. (2012), where the PET (polyethylene terephthalate) non-woven fabric was coated with zirconium dioxide (ZrO_2_) fine particles prior to the deposition of AuNPs to enhance the catalytic activity. An in situ reaction method was used to produce and deposit AuNPs by the deposition-precipitation method [57]. Deshan Cheng et al. (2019) synthesized AuNPs via in situ synthesis reduction method and deposited them on cotton fabric by dip-coating. In this work, HAuCl_4_ was used as the precursor salt and polydopamine (PDA) acted as reducing agent. AgNPs were deposited prior to the deposition of AuNPs, which acted as catalytic hotspots for enhancing the deposition of AuNPs [58]. Hongjun Liu et al. (2013) used HAuCl_4_ to synthesize AuNPs on cotton fibers via in situ synthesis using the amine stabilizers and N-vinyl pyrrolidone (PVP) reductant in an aqueous medium. Fibers of cotton surface were first modified with (3-aminopropyl) triethoxy silane (ATS). The pre-treatment of the surface was found to be essential, without which a small amount of nonuniformly adsorbed AuNPs was formed on the fabric. The size of the nanoparticles was in the range of 2–7 nm and attained spherical shape [59]. Bharat Baruah et al. (2019) have focused on improving the catalytic activity of the fabrics combining zinc oxide (ZnO) and AuNPs. Hence, the fabric was coated with ZnO nanorods prior to the deposition of AuNPs. AuNPs was obtained by ex situ synthesis and citrate reduction method. AuNPs of the size 18.5 ± 2.8 nm were then deposited on cotton fabric coated with ZnO nanorods by the dip-coating method [60]. Hongjuan Ma et al. (2013) have functionalized PE/PP non-woven fabric by gold nanostructured microtubes, where AuNPs were in situ prepared after the grafting of 4-hydroxybutyl acrylate glycidyl ether onto the shell PE layer and posterior reaction with diethylamine. The tertiary amine groups covalently bonded onto the fabric are designed as the adsorbent for metal ions and reducing agent. Spherical-shaped nanoparticles with size of 5–20 nm were obtained in this study [61]. Richard P. Padbury et al. (2015) produced 14.6 ± 1.4 nm gold nanoparticles using HAuCl_4_ as precursor salt. The sheets made up of nonwoven nylon-6 were soaked in NP solution and AuNPs are settled on the fabric [62]. It was observed that size and shape of nanoparticles depend upon reducing agent and concentration of the solution. These strategies produced functional textiles with improved properties, opening new perspectives for studies using combined approaches, able to provide the maximum AuNPs effect using low concentrations. Thus, improved methods may be obtained with reduced costs and environmental impact.

#### 4.1.3. Chemical Reduction Method Using Thermal Treatment

Some works were found in the literature that described the potential of functional groups in the composition of fibers to act as reducing agents of gold salts under heating. These works synthesized AuNPs in situ of silk fibers due the presence of tyrosine amino acid. Bin Tang et al. (2014) functionalized the pristine silk fabric by in situ synthesis of AuNPs. The silk fabrics with tetracholoroaurate ion (AuCl_4_^−^) were heated at 85 °C in solution, and the AuNPs were in situ synthesized on silk fabric. Nanoparticles of size 21.3 ± 3.4 nm and different shapes were observed, and it was noticed that the concentration of the precursor in solution have influenced the shape of the nanoparticles and also improved thermal conductivity [63]. Jun Liu et al. (2016) have produced silk fabrics coated with gold nanoparticles. Gold nanoparticles were in situ synthesized on silk fabrics by heating precursor salt (HAuCl_4_.3H_2_O). The AuNPs were deposited on the silk fabrics by immersion of fabrics in the solution and heating the solution at 85 ℃ for 60 min. Concentration of the precursor solution had an influence on the shape of the AuNPs and attained spherical, triangular nanoplates, truncated nano prisms, and polygonal shapes with varying concentrations [64]. Zhanyu Zhang et al. (2019) have synthesized AuNPs from HAuCl_4_ by in situ process and maintaining pH value at 3. AuNPs coated the silk fabrics by immersion of fabric in solution and applying heat process [65]. These works showed the importance of functional groups in the fiber composition and how they can be used to obtain functional textiles and suggested novel studies using other protein fibers.

#### 4.1.4. Green-Bio Synthesis

To facilitate the use of gold nanoparticles in the health sector and minimize the environmental impact, it is essential not to use any toxic or harmful chemical during the synthesis. Hence, researchers have been shifted towards green synthesis methods, where the reducing and stabilizing agents are obtained from plants or a few at times bacteria. All the works that evolve biological methods for AuNPs are quite recent, being this approach a high research tendency. R.M. Ganesan et al. (2015) have synthesized AuNPs using HAuCl_4_ as a precursor and extract of *Acorus calamus rhizome* as a reducing agent. Then, the cotton fabrics were coated by the pad-dry-cure method. The synthesized AuNPs were small and big spherical shape and had different sizes depending upon concentration of the solution [66]. Nabil A. Ibrahim et al. (2016) have biosynthesized AuNPs using HAuCl_4_3H_2_O as a precursor salt and bacterial isolates *(Streptomyces sp.)* as a reducing agent. The surfaces of cotton and viscose knitted fabrics were modified using plasma treatment before the functionalization with gold nanoparticles in combination with TiO_2_NPs or ZnONPs. The AuNPs were observed to have spherical shape with size in the range of 4–13 nm. The nanoparticles were deposited on to the knitted fabrics by sonication [67]. Bin Tang et al. (2017) have used in situ synthesis method to prepare AuNPs onto a cotton fabric using HAuCl_4_ solutions at the concentrations of 0.025, 0.05, 0.075, 0.10, and 0.125 mM. Cellulose acted as reducing and stabilizing agents. The obtained AuNPs have exhibited different shapes depending on the content of gold such as spherical and triangular nanoplates with different sizes. The coated fabric displayed catalytic activity and improved UV protection [68]. To obtain a green approach, Jinlon Tao et al. (2018) functionalized cotton and polyester fabric by hybrid colloids of AuNPs and NRP (natural rubber particles), which was obtained through in situ synthesis of AuNPs in NRL (natural rubber latex) matrix. In this process, NRL act as both reducing and capping agent. HAuCl_4_ solution and NRL was employed for obtaining AuNP@ NRP hybrid latex. The team induced the hierarchical nature into the material by the addition of NRL, which lead to the phenomena of hydrophobicity of the treated fabrics. The fabric surface was coated with AuNPs of size 31 nm using the dip and dry method [69]. P. Boomi et al. (2019) have functionalized cotton fabric using gold nanoparticles produced by green synthesis reduction method by maintaining pH value equal to 7. In this study, AuNPs were synthesized by reducing HAuCl_4_ with *Coleus aromaticus* leaf extract. The AuNPs were coated on the cotton fabric by immersion of the fabric in the colloidal solution. The obtained nanoparticles were of spherical and triangular shape and different sizes were measured [70]. Pandi Boomi et al. (2020) have synthesized AuNPs and deposited them on the cotton fabric to improve their antibacterial and anticancer properties. The gold nanoparticles were synthesized by green synthesis, where *Croton sparsiflorus* leaves extract acted as both reducing and stabilizing agent and HAuCl_4_ as precursor salt. The cotton fabric was coated with pristine leaf extract through the pad-dry-cure method. Different sizes between 16.6 and 17 nm were obtained using high concentration and low concentration solution, respectively [71]. Simone Haslinger et al. (2019) reported a novel strategy that was attempted for the first time. Noble metal nanoparticles were added into the cellulose pulp by a hydrothermal approach and subsequently subjected to dry-wet spin process. They have functionalized cellulose-based textiles with Au and AgNPs. In this study, bleached *birch prehydrolyzed kraft pulp* acted as a reducing agent and HAuCl_4_ as precursor salt to synthesize AuNPs. These nanoparticles were incorporated into the textile by dry-jet wet spinning process, which improved the UV protection and helped to achieve bright colors [72]. Some attempts to use the combination of both chemical and green methodologies to study the synergetic effect of both methodologies have been performed. Velmurugan et al. (2016) synthesized AuNPs using in situ synthesis method onto leather, silk, and cotton fabrics by three different methods that include green, chemical, and a combination of green and chemical synthesis. *Ginkgo biloba* Linn leaf powder extract, HAuCl_4_, and potassium borohydride (KBH_4_) were used in green and chemical synthesis. For the combination of green and chemical synthesis, *Ginkgo biloba* Linn leaf powder extract and KBH_4_ were used, and the obtained nanoparticles were deposited by immersion of the fabrics in the solution. TEM observations had revealed nanoparticles in the range of 10–75 nm with either rectangular, spherical, hexagonal with smooth edges, or roughly circular in shape [73]. The use of biological methods showed several advantages, but more studies are needed to solve reproducibility issues, understand the influence of AuNPs attached groups in the assigned properties, and implement them at the commercial level.

#### 4.1.5. Electrochemical Synthesis

Electrochemical synthesis is a simple and inexpensive method to obtain NPs but a few studies were found in the literature in the textile field. Mauro Pasta et al. (2012) have produced a conductive textile by coating single-walled carbon nanotubes on a polyester textile substrate. Subsequently, AuNPs were prepared using HAuCl_3_ in the presence of HCl electrolyte. Later, AuNPs were electrodeposited onto the conductive textiles and the size of the nanoparticles was found with a mean diameter of 50 nm [76].

### 4.2. Functionalization of Fibers/Yarns/Threads with AuNPs

Similarly, to the fabrics, the functionalization of fibers, yarns, and threads is performed mostly using chemical methods. A few reports were found in the literature using the other strategies (Table 2). AuNPs were deposited in these textile materials by commonly used methods, namely, exhaustion, immersion, soaking, sonication, and electrodeposition. Here, the use of most recent approaches such as in situ and biological methods to prepare AuNPs are limited and numerous researcher studies can be developed.

#### 4.2.1. Chemical Reduction Method without Pre-Treatments on Fabrics

David R. Ballerini et al. (2014) have synthesized AuNPs by the Turkevich method using HAuCl_4_ as precursor salt and sodium citrate tribasic dihydrate as a reducing agent. AuNPs of size of 20 and 60 nm were obtained and deposited on the cotton thread by soaking them individually in the solution of AuNPs. The cotton threads were treated with cationic polyacrylamide (CPAM) before the deposition of AuNPs [77]. Cristina Battesini Adamo et al. (2020) have synthesized spherical-shaped gold nanoparticles of size 20–40 nm using the Turkevich method. In this study, HAuCl_4_ solution (30 wt%) and sodium citrate were used. The AuNPs were wicked on to the cotton thread by capillary action when threads were immersed in the solution [78]. Md. Tariqul Islam et al. (2016) have produced AuNPs with average size of 21.01 nm where HAuCl_4_ was used as a precursor salt and sodium squarate in water was used as a reducing and stabilizing agent. The gold nanoparticles were synthesized in water and attached to the cellulose fibers by sonication [79]. Hyung Ju Park et al. (2016) have fabricated cotton yarns decorated with Au core-shell nanoparticle by a solution-based approach. The AuNPs were synthesized by a chemical reduction method where HAuCl_4_ was used as a precursor salt solution and trisodium citrate dihydrate was used as a reducing agent and stabilizing agent. The solution was maintained at pH between 3 and 4, and the obtained nanoparticles were of the size 13 nm [80]. 

Some reports conducted with in situ methods have been reported, but in all studies, additional chemical reducing agents were used. Youvi Xia et al. (2011) have produced sulfonated polyaniline-modified silk fibroin fibers coated with AuNPs. AuNPs of size in the range of 50–100 nm were synthesized using HAuCl_4_ via in situ reduction method where sulfonated polyaniline acted as reducing agent. They were deposited by soaking fibers in the solution that contains AuNPs [81]. Bin Tang et al. (2015) have synthesized AuNPs through in situ synthesis using HAuCl_4_ with different concentrations and NaBH_4_. The pH value of the solution was maintained such that it had acidic condition. They were deposited on to the ramie fibers by immersion of fibers in the solution [82]. Tariqul Islam et al. (2017) have used in situ synthesis where HAuCl_4_ was used as precursor salt and sodium rhodizonate was used as a reducing and stabilizing agent to produce AuNPs. The AuNPs were found to have spherical shape and the size of 7 and 11 nm. The stabilized AuNPs were readily adsorbed on cellulose fibers [83]. Hossam E. Emam et al. (2017) have used one-pot fabrication of AgNPs onto a cellulosic solid support. AuNPs of 26.1 nm were synthesized through green synthesis using gold chloride (AuCl_3_), NaBH_4_ as reducing agent, and cellulosic macromolecules as stabilizing agent. They were deposited onto cellulose solid support by one-step method [84]. Yong Ju Yun et al. (2017) have fabricated gold/graphene yarns using a solution-based process to potentially use them for flexible and wearable electronics. HAuCl_4_ was used as precursor salt and hydroxylamine was used as a reduced agent for the synthesis of AuNPs. They were deposited uniformly on the surface by electroless deposition and found to have from spherical shape to plate and grow vertically with time, the size of the AuNPs was 40 nm in diameter [85]. Qian Yu et al. (2018) have produced multifunctional cellulose fiber Au composites via decorating regenerated cellulose fiber with AuNPs. HAuCl_4_ was used as a precursor salt and trisodium citrate was used as a reducing and stabilizing agent to produce gold nanoparticles following Natan’s method. The obtained spherical AuNPs with size of 40–50 nm were decorated on to the surface of cellulose fiber by the immersion of fibers in the solution of Au colloids. The fibers were grafted with positive charge according to the method proposed by Tabba and co-workers with some modifications [86].

#### 4.2.2. Chemical Reduction Method with Pre-Treatments 

Yan Liu et al. (2017) produced AuNPs with size of 15 nm using HAuCl_4_ solution and trisodium citrate by chemical reduction method. AuNPs were coated on the CNTs, which were priorly functionalized with poly(diallyldimethylammonium chloride) (PDDA) using sonication method, and these were deposited on to the cotton thread by soaking cotton threads in the solution that contains AuNPs/CNTs. To improve the wicking function of the cotton thread, chemical treatments are necessary to eliminate the surface contaminants [87]. Xiaobo Jia et al. (2017) have synthesized AuNPs with size of 15 ± 3 nm using HAuCl_4_ solution and trisodium citrate by chemical reduction method. These nanoparticles are mixed with CNT and subsequently, the cotton thread device was constructed for carcinoembryonic antigen (CEA) detection. It was constructed by soaking the cotton thread in the solution containing AuNPs-coated CNTs that were previously functionalized with PDAA [88].

#### 4.2.3. Reduction with Photochemical Treatments

Bin Tang et al. (2011) have synthesized AuNPs using HAuCl_4_ as precursor salt and citrate, malate, and tartrate as reducing agents by heating and photochemistry processes. The solution was used to color silk and nylon fibers, and AuNPs were deposited using the exhaustion process. Nanoparticles were observed to have egg, spherical shape and various sizes depending on the type of reducing agent [75].

#### 4.2.4. Green Synthesis

Naseeb Ullah et al. (2019) have used HAuCl_4_ as a precursor salt and *Osmanthus fragrans* leaves as reducing and capping agent irradiated by natural sunlight to produce AuNPs. The cotton fibers were then immersed in the AuNPs solution, and they were deposited on the fabrics through in situ reduction in the presence of direct sunlight. Nanoparticles of size 40 and 60 nm in diameter were observed in TEM and appeared to have spherical and hexagonal shapes [89]. Victor Nolasco-Arizmendi et al. (2012) have synthesized AuNPs using HAuCl_4_ as the base solution and *citrus paradise* extract as the reducing agent, and the AuNPs were deposited on the silk fabric by impregnating the fabric in the solution. Quasi-spherical-shaped nanoparticles were observed [90].

### 4.3. Functionalization of Nanofibers/Scaffolds/Membranes with AuNPs

The progress in synthetic or artificial fibers has created several opportunities to develop novel multifunctional textiles. Spinning methods, particularly, the electrospinning, have provided fibers from different polymers and have been widely used to obtain nanofibers and scaffolds conjugated with AuNPs. Very limited literature is available in recent years that used green synthesis methods to produce AuNPs and deposited them on nanofibers/scaffolds/membranes. In the case of chemical methods, most studies perform the in situ AuNPs synthesis directly in the electrospinning solution. In addition, in very specific cases, the AuNPs are previously synthesized and mixed in the solution before electrospinning. In the case of membrane functionalization, the AuNPs are deposited by soaking or immersion (Table 3).

#### 4.3.1. Chemical Reduction Method without Pre-Treatments

The two-step chemical reduction method, the AuNPs synthesis and posterior textiles functionalization, was widely applied. Wei Wang et al. (2010) have produced AuNPs–Bacterial cellulose (BC) nanocomposites. In this study, AuNPs were synthesized by the reduction of HAuCl_4_ in the presence of NaBH_4_ that acted as reducing agent, mixed with BC nanofibers. Produced AuNPs have measured approximately 9 nm. The AuNPs were deposited on fibers by immersing them in the solution of BC nanofibers and applying ultrasonic cell disruption system at room temperature. [93]. Hirotaka Koga et al. (2010) have synthesized AuNPs on crystalline cellulose single nanofibers (CSNFs). Preparation of AuNPs includes the addition of CSNFs in the solution of HAuCl_4_ followed by reduction with NaBH_4_. The nanoparticles of size less than 5 nm were deposited because of immersion of fibers in nanoparticles in solution [91]. Taiji Zhang et al. (2010) have obtained spherical-shaped AuNPs of approximately 9 nm by the reduction of HAuCl_4_ in the presence of polyethyleneimine (PEI) that played the role of both reducing and stabilizing agent, and it also acted as a linking agent to uniformly coat the AuNPs on to the BC nanofibers. AuNPs were coated on to the BC nanofibers by suspending them in the solution [92]. Bin Zhou et al. (2014) have produced antibacterial multilayer films coated with gold nanoparticles on nanofibers. Gold nanoparticles were synthesized using chemical reduction method. HAuCl_4_ solution and trisodium citrate were used as precursor salt, reducing and stabilizing agent for the synthesis of AuNPs, and they were coated on to the electrospun cellulose acetate fiber mats by immersion of mats into colloidal gold nanoparticles solution [94].

Hui-Hui Cheng et al. (2016) have fabricated thermoplastic polyurethane electrospun fiber mat and deposited the gold nanoparticles after the functionalization of the mat with chitosan by dip-coating method. The AuNPs of size 16 nm were produced using HAuCl_4_, trisodium citrate as reducing agent, and chitosan as stabilizing agent [95]. Xinglong Yang et al. (2017) have produced antibiotic intermediate capped AuNPs by chemical reduction of HAuCl_4_ by NaBH_4_. The process was done in the presence of antibiotic intermediates. These nanoparticles of size approximately 3 nm have been embedded on to the polycaprolactone (PCL)/gelatin nanofibers in the form of scaffolds during electrospinning [96]. Ying Li et al. (2017) have produced a bacterial cellulose (BC) membrane decorated by AuNPs and modified with 4,6-diamino-2-pyrimidinethiol (DAPT) for treating bacterially infected wounds. In this research work, AuNPs of size 3 nm were produced via in situ synthesis method where HAuCl_4_ and DAPT solution was used. The AuNPs were deposited on to the BC membranes by soaking them in the solution [97]. Xu Fang et al. (2010) have produced electrospun poly(ethyleneimine)/polyvinyl alcohol nanofibers and immobilized them with round-shaped gold nanoparticles of size 11.8 nm. In this study, the AuNPs were synthesized and deposited on to the electrospun fibers via in situ reduction of AuCl_4_^−^ ions using NaBH_4_ as reducing agent [98]. Zhen Liu et al. (2011) have produced electrospun porous polyacrylonitrile nanofibers and functionalized them with AuNPs. AuNPs were synthesized by in situ reduction of HAuCl_4_ to Au (0) by 4-(dimethylamino) benzaldehyde. The electrospun membrane was immersed into the buffered hydrogen tetrachloroaurate (III) trihydrate solution and then, AuNPs of size 6 nm, were adsorbed onto the nanofibers [104]. Anitha Senthamizhan et al. (2014) have produced DTT capped AuNPs by in situ synthesis using the gold solution and dithiothreitol. AuNPs of the size 2.5 ± 0.5 nm were deposited on the cellulose acetate fibrous membranes by soaking them in the solution [105].

Xiaodong Chen et al. (2014) have functionalized zein ultrafine fibers by AuNPs. The zein ultrafine fibers were produced by electrospinning and, subsequently, AuNPs were added on to the fibers by one step reduction method (in situ polymerization) using HAuCl_4_ as precursor salt and poly(ethyleneimine) (PEI) as reducing agent and cross-linking agent. The pH was maintained between 3 and 7, and the size of the AuNPs were observed to be 90.9 nm in diameter [106]. Han Zhu et al. (2014) have synthesized AuNPs embedded polyacrylonitrile nanofibers by the combination of in situ reaction and electrospinning. AuNPs were produced using the HAuCl_4_ solution via in situ reduction method. The nanofibrous mats with AuNPs embedded in it were achieved by the electrospinning technique and spherical-shaped AuNPs with size of 2.3 ± 0.5 nm [107]. Diana Serbezeanu et al. (2015) have produced gold-containing polyimide fibers. AuNPs were synthesized using an in situ electrospinning approach where HAuCl_4_.3H_2_O was used as precursor salt and polyimide was used as stabilizing agent. The reduction takes place due to the thermal reactions and the SEM observations displayed nanoparticles of the size 9–22 nm [108]. Simón Yobanny Reyes-López et al. (2015) have proposed a facile method to produce gold-coated polycaprolactone nanofibers. Reduction method was employed to synthesize AuNPs, round-shaped 5–6 nm nanoparticles, using HAuCl_4_ and NaBH_4_ as a reducing agent. The gold nanoparticles were separated in a rotavapor, were added into the viscous solution of PCL, and subjected to electrospinning [99].

#### 4.3.2. Chemical Reduction Method with Pre-Treatments 

Tao Niu et al. (2014) have produced cellulose-based membranes and coated the membranes with AuNPs. In this research study, AuNPs were synthesized with KAuCl_4_ and NaBH_4_ and these nanoparticles were deposited on membranes previously coated with Titania gel [109]. Riyas Subair et al. (2016) synthesized gold nanoparticles on the PET track-etched micro-porous membranes. Before synthesis on nanoparticles, these membranes were coated with dopamine and poly(ethyleneimine). The authors followed two types of methodologies for the synthesis of AuNPs. AuNPs were synthesized by self-reduction of AuCl_4_^−^ immobilized on the surface of dopamine membrane, and AuNPs were synthesized via in situ reduction with NaBH_4_ on PEI membranes. In both the cases, AuNPs were deposited by immersion of the membranes into nanoparticles solution and maintaining continuous shaking condition [100]. Mohammed Awad Abedalwafa et al. (2019) have functionalized polyamide nanofiber membranes using AuNPs. The AuNPs were synthesized via citrate reduction technique where HAuCl_4_ and trisodium citrate dihydrate were used as precursor salt and reducing agent, respectively. AuNPs were functionalized with melamine (MA) and were immobilized on polyamide 6 nanofiber electrospun membranes by dropping MA@AuNPs on the membranes. AuNPs and MA@AuNPs were found to have the size of 20.1 ± 1.7 and 28.4 ± 2.3 nm, respectively [101]. Yan Chen et al. (2016) have deposited AuNPs on bacterial cellulose nanofibers treated with 2,2,3,3-tetramethylpiperidine-l-oxyl (TEMPO)-oxidized (TOBCNS). AuNPs were produced by chemical reduction method using HAuCl_4_ and NaBH_4_, and simultaneously, they were deposited on to the TOBCNS by immersing nanofibers in the solution. It was observed from TEM that nanoparticles were spherical having size of 4.30 ± 0.97 nm [102].

#### 4.3.3. Chemical Reduction by Photo Reduction/UV Radiation/Photo Reduction

Fadwa H. Anka et al. (2012) have produced polyacrylonitrile nanofibers using electrospinning, and they have been functionalized with AuNPs. The AuNPs were synthesized via in situ photoreduction. In this study, HAuCl_4_ was the precursor salt and sodium alginate acted as reducing agent. UV light has been used during electrospinning. Spherical shape was observed with a size of 21.4 and 5.8 nm [111]. Koichi Sawada et al. (2013) have produced PAN (Polyacrylonitrile) nanofibrous fabrics and deposited AuNPs onto the fabrics. PAN fabrics were produced by electrospinning of PAN solution and HAuCl_4_. AuNPs were formed on the fabrics by in situ gold formation using UV irradiation. The average size of the gold nanoparticles obtained was 4.7–5.4 nm [112].

Xiaodong Wu et al. (2014) have used one-pot and green synthesis to synthesize and deposit the gold nanoparticles on to cellulose nanocrystals. By means of hydrothermal reaction AuNPs were deposited on to the cellulose nanocrystals. In this work, they have used HAuCl_4_ solution and cellulose nanocrystals as a both reducing and stabilizing agent to synthesize AuNPs with size of 30.5 ± 13.4 nm. These cellulose nanocrystals are derived from cotton fabrics. In the same study, unsupported AuNPs were synthesized, using hydrazine hydrate as reducing agent, with size of 18.3 ± 3.5 nm [103]. Xu Zhou et al. (2018) have synthesized AuNPs on BC using the photoinduction method. In this study, HAuCl_4_ was used as precursor salt and xenon lamp was used as reducing agent. AuNPs were deposited by immersing the BC hydrogels in the HAuCl_4_ solution [113].

#### 4.3.4. Green Synthesis

Moeng G. Motitswe et al. (2020) have used the green technique for the synthesis of AuNPs where banana (*Musa paradisiaca*) was used as a reducing agent and HAuCl_4_ was used as the precursor salt. The spherical-shaped 9 nm AuNPs were deposited on to the polyacrylonitrile nanofibers using electrospinning technology [2].

#### 4.3.5. Reduction with Thermal Treatments

Maggalena Aflori et al. (2015) have incorporated gold nanoparticles into electrospun polyimide fibers. In this work, HAuCl_4_ was used as gold precursor and treated thermally at 200 °C for 6 h to reduce Au^3+^ to Au^0^. The electrospinning technique was used to incorporate the AuNPs into polyimide fibers [18]. 

#### 4.3.6. Reduction with Other Treatments

Amir Shahin Shamsabadi et al. (2019) have produced AuNPs by laser ablation in the solution of PAN in dimethyl formamide. After that, spherical-shaped AuNPs were mixed with the PAN solution to subject to electrospinning and produce PAN nanofibers incorporated with AuNPs [114].

## 5. Antimicrobial Properties of AuNPs on Textiles

The demand for the development of materials with antimicrobial properties has driven different studies encompassing AuNPs and textile materials. Several methodologies have been developed to improve their activity. However, an endless number of studies can be conducted considering the promising current results. As mentioned above, the antimicrobial effect of AuNPs is dependent on different physicochemical properties. In addition, several studies demonstrated an improved effect of some antibacterial agents in the presence of AuNPs [115]. Thus, the development of new methods to obtain antimicrobial textiles based on AuNPs is worth studying. Here, the main antimicrobial results of textile materials functionalized with AuNPs are presented (Table 4 and Table 5).

The textile materials used in these studies were mainly fabrics or knitted fabrics of natural fibers (cotton, silk, wool, and soybean) or artificial fibers (viscose); a few studies exist with synthetic fibers. Considering the microorganisms to test the antimicrobial efficacy, some strains were used, such as (i) Gram-positive *Staphylococcus aureus* (*S. aureus*) was frequently tested but also the effect against *Staphylococcus epidermidis* (*S. epidermidis*) and *Bacillus cereus* (*B. cereus*) were studied and (ii) belonging to Gram-negative strains, the *Escherichia coli* (*E. coli*) was the most studied but also reports using *Klebsiella pneumoniae* (*K. pneumoniae*) and *Pseudomonas aeruginosa* (*P. aeruginosa*) were found; just one work was found using yeasts, the *Candida utilis* (*C. utilis*). Based on the results, the addition of AuNPs to textile materials enhanced their antimicrobial properties. The studies can be divided into two types, one using just AuNPs for textiles functionalization and another where the AuNPs were conjugated with other agents. A few studies were found in which only the functionalization of textile materials was made with AuNPs. In all cases, the antimicrobial effect is noticed but the effect is mainly bacteriostatic and/or by contact. A few studies revealed bacterial reduction only using AuNPs. Conjugation of AuNPs with other agents is more frequent and AuNPs have been combined with chitosan, antibiotics, other NPs (Ag, Pt and ZnO), plant extracts (that act both as reducing agent and also present antimicrobial effect), and antimicrobial enzymes (lysozyme). In these studies, the conjugation significantly increased the antimicrobial activity than the use of each of the components separately. In addition, even molecules that do not show considerable antimicrobial activity before the conjugation, when combined with AuNPs, became highly active in textile composites.

AuNPs were added to the plasma-treated polypropylene fabric by Nina Radic et al. (2012). Agar diffusion test was used to test those fabrics for antibacterial activity. Two types of organisms were used Gram-positive (*S. aureus,* ATCC 6538) and Gram-negative (*E. coli,* ATCC 11229). The results showed that both of the plasma-treated fabrics have shown similar kind of antibacterial activity against Gram-positive (*S. aureus*) and Gram-negative bacteria (*E. coli*). The fabrics presented antimicrobial activity in contact. It was observed that *S. aureus* was more sensitive to the AuNPs-loaded polypropylene fabrics. The best antibacterial activity was shown in the fabric pre-treated for 12 s (24 J/cm^2^) by diffuse coplanar surface barrier discharge (DCSBD) and 120 s (14.4 J/cm^2^) by dielectric barrier discharge (DBD) [56]. Functionalized and untreated silk fabrics were tested for antibacterial properties by Bin Tan et al. (2014) according to AATCC 100-2004 (Clause 10.2) test standard with slight modification. Gram-negative bacteria (*E. coli*, ATCC 11229) were used for performing the test, and they found that there was no presence of *E. coli* bacteria colonies after the contact. This observation proved that gold nanoparticles have inhibited the growth of bacteria [63]. Bin Zhou et al. (2014) have produced antibacterial multilayer films of cellulose acetate conjugating AuNPs and lysozyme, an antimicrobial enzyme, and the films were tested for antibacterial activity using *S. aureus* and *E. coli*. An inhibition zone test was used, and the composite mats have revealed significant antibacterial activity. The inhibition activity was better against *S. aureus* than against *E. coli* [94]. Cotton fabrics coated with AuNPs were tested for antibacterial activity against *S. aureus* (MTCC 96) and *E. coli* (MTCC 1671) by RM Ganesan et al. (2015) according to AATCC 100 test method. The results prevailed that higher antibacterial activity was shown against *E. coli* than *S. aureus*. Reduction in 50.05% and 63.9% against *S. aureus* after 24 and 48 h culture, respectively, and reduction in 58.0% and 80.3% against *E. coli* after 24 and 48 h culture, respectively, was observed [66]. 

The agar plate method was used by Nabil A Ibrahhim et al. (2015) to determine the antibacterial activities of AuNPs and AuNPs-ZnONPs loaded viscose and cotton substrates. They have used Gram-positive bacteria (*S. aureus*) and Gram-negative bacteria (*E. coli*). It was observed that the plasma-treated cotton and viscose knitted fabrics have shown good antibacterial activity against both bacteria. The highest activity was shown with the plasma-treated AuNPs in combination with ZnONPs for both, where cotton presented an inhibition zone against *E. Coli* of 27.5 and 30 mm for *S. aureus* and viscose fabrics showed an inhibition zone against *E. Coli* of 25 and 27 mm for *S. aureus* [67]. Gram-negative bacteria *E. coli* (ATCC 11229) was used by Bing Tang et al. (2017) for testing the antibacterial activity of a knitted cotton fabric coated with gold nanoparticles. The test was carried out according to AATCC 100-2004 (clause 10.2) standard with slight modification. The results indicated that the presence of AuNPs on the treated cotton fabric inhibited the growth of bacteria when compared to the untreated samples [68]. The antibacterial activity of the nanogold-coated silk fabric produced by Hanan B Ahmed et al. (2017) was tested using *E. coli* (ATCC- 25922) and *S. aureus* (ATCC 47077). The bacterial growth was estimated by the turbidimetric method, and the results indicated that the antibacterial activity was enhanced with the deposition of AuNPs. The optical density of *E. coli* and *S. aureus* was reduced from 2.55 to 1.11–1.23 and from 2.12 to 0.93–1.1 after 24 h incubation, respectively [54]. Ying Li et al. (2017) have used the shake flask test method to determine the antibacterial performance of bacterial cellulose (BC) membrane decorated by AuNPs modified with 4,6-diamino-2-pyrimidinethiol (DAPT). In this study, *E. coli* (ATCC 1175), *P. aeruginosa* (1.2387), MDR E. coli (Q1124), and MDR *P. aeruginosa* (N3966) were used, and it was observed that BC-Au-DAPT nanocomposites inhibit bacterial growth and promote wound repair [97]. Elmaaty et al. (2018) finished polyester and cotton fabrics using AuNPs, the samples exhibited excellent antimicrobial activities against *S. aureus, B. cereus, E. coli,* and *C. utilis* [42]. O Shanmugasundaram et al. (2018) used Kirby–Bauer test methods for determining the level of inhibition of gold nanoparticles along with keratin protein. The results showed that they have a strong inhibition effect against bacteria *K. pneumoniae* followed by *P. aeruginosa*, *S. aureus*, and *E. coli*. The study reported zone diameters ranging from 3.2 cm for *E. coli* and 4.4 cm for *K. pneumoniae* [49]. Lin Zhou et al. (2019) have used *S. aureus* and *E. coli* strains for detecting the antibacterial performance of dressing fabrics (woven silk fabrics) by broth microdilution method and were measured via inhibition zone method. The results have shown that AuNPs alone do not have a significant effect but when they are used in combination with gentamicin sulfate (GM), the fabrics showed stronger antibacterial effects (inhibition zone against *E. coli* and *S. aureus* was 25.8 and 26 mm, respectively) when compared with the usage of GM alone on the silk fabric [55].

The silk fabrics, which were functionalized with AuNPs by Zhanyu Zhang et al. (2019), were tested using the quantitative microbial colony forming units (CFU) method for *E. coli* (ATCC 25922). The tests were conducted following AATCC 100-2012 (Clause 10.2) standard with slight modifications. The results showed that the values are close to 99.6% for the functionalized silk fabrics after complex coloration and the addition of traditional dyes did not hinder the antibacterial effect of AuNPs [65]. Iris O. Silva et al. (2019) functionalized chitosan-treated and untreated soybean knitted fabrics with AuNPs and assessed their antimicrobial properties following the shake flask method (ASTM-E2149-01). In this test, both *S. aureus* and *E. coli* were used, and it was found that the soybean knitted fabric treated with chitosan and functionalized with AuNPs was most efficient in fighting the bacteria with the reduction in 99.94 against *S. aureus* and 96.26% against *E. coli* after 24 h culture [52]. Boomi et al. (2019) have functionalized cotton fabric with AuNPs, and the fabric was tested against *S. aureus* and *E. coli*. It was observed that the coated fabric exhibited inhibition zones for both bacteria. The inhibition zone against *E. coli* and *S. Epidermidis* was found to be 27 and 22 mm, respectively [70]. Pandi Boomi et al. (2020) have studied the antibacterial activity of cotton fabric functionalized with gold nanoparticles and found that the coating improved the antibacterial activity of these fabrics. The cotton fabrics were tested against *E. coli* (MTCC 10546) and *S. epidermidis* (MTCC 35984) by disk diffusion susceptibility assay. The results showed an inhibition zone of 30 and 26 mm diameter against *S. epidermidis* and *E. coli,* respectively [71].

**Figure 4:**
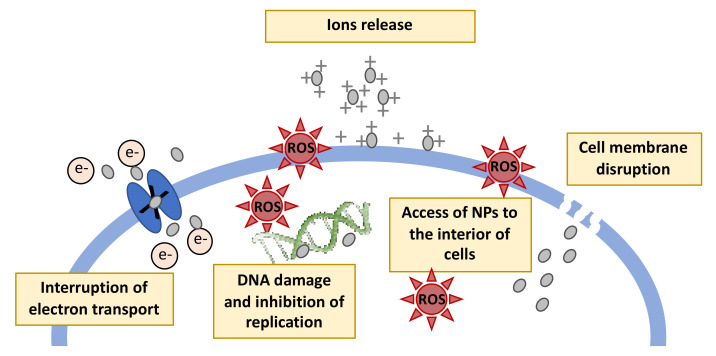
Mechanisms of antimicrobial action of the metal nanoparticles. (Adapted from [116]).

Several studies have been performed to better understand the AuNPs antimicrobial mechanism of action. Generally, metal nanoparticles can interfere with bacteria in different ways, namely, generating reactive oxygen species (ROS), disrupting the cell wall, interrupting the transport of electrons, and inhibiting the DNA replications (Figure 4) [116]. Particularly, AuNPs antibacterial effect showed to act in two steps: first is the collapse of the membrane potential, inhibiting ATPase activities to decrease the ATP level, and second is the inhibition of subunit of ribosome from binding tRNA. AuNPs increase chemotaxis in the early-phase reaction. The antimicrobial effect of AuNPs did not comprise ROS generation, the cause for cellular death induced by most bactericidal antibiotics and nanoparticles [117]. Additionally, the small size and high surface area also increase the surface reactivity of NPs, and they can get in contact with the bacteria in an efficient way. The functions of the bacterial cell wall depend on the proteins and the cytoplasm and the functioning of the proteins will be affected by the nanoparticles and hence the cell death will occur. The tendency of the AuNPs to react with the sulfur of phosphorous groups will make the DNA molecule a prominent target to attack by AuNPs. Concentration and size of AuNPs are the most influential parameters for the antibacterial effect and it is linearly proportional to the average size of AuNPs [7].

**Figure 5:**
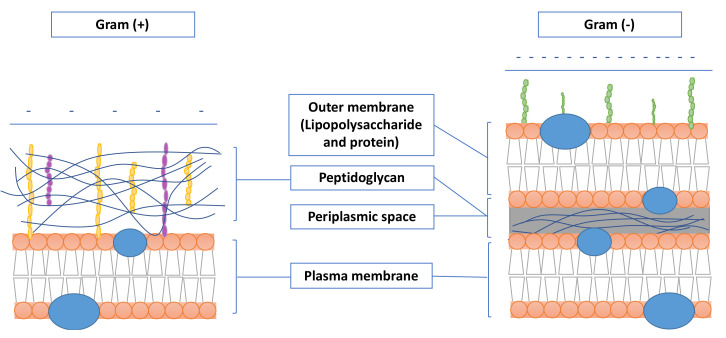
Difference between Gram-positive and Gram-negative bacteria (Adapted from [119]).

The antibacterial effect of textile materials functionalized with AuNPs showed to be more effective against Gram-positive than Gram-negative bacteria, and this may be related to the differences in the structure of Gram-positive and Gram-negative bacteria (Figure 5). Both of them have a negatively charged surface but the thickness of the peptidoglycan layer and presence or absence of the outer lipid membrane can induce relevant disparities in the antimicrobial action of the AuNPs. Gram-positive bacteria have a thick layer of peptidoglycan formed by linear chains, forming a cohesive mesh. On the other hand, Gram-negative bacteria, have a slightly more complex structure. Besides the thin layer of peptidoglycan, Gram-negative bacteria have a phospholipid outer membrane with partially phosphorylated lipopolysaccharides that provide an increased negative surface charge. In this way, bacterial cell walls attract positively charged nanoparticles to their surface due to electrostatic interactions. They establish a strong bond with membranes, resulting in disruption of cell walls, increasing their permeability. Gold ions can form strong coordination bonds with N, O, or S atoms, which promote a broad-spectrum activity [118]. 

## 6. Conclusions with Future Perspectives

This review aimed to underline the scientific literature comprising AuNPs and textile materials, as well as to highlight those that present antimicrobial properties. To this end, two crucial points were identified for the development of these materials. The main factors that constrain their commercial use and adaptation to large-scale processes were found to be related to the AuNPs synthesis and the methods to their deposition. Regarding the synthesis of nanoparticles, chemical methods are still the most used and guarantee greater control of the physical–chemical properties of the obtained NPs. However, due to its high toxicity and cost, methods for its simplification and replacement have been progressively studied. In the deposition of AuNPs onto textile subtracts, two distinct approaches were identified. The first one is the synthesis of AuNPs and their subsequent deposition, and the second one consists of in situ synthesis of AuNPs, replacing a two-step by one-step process. The in situ method has shown a reduction in the time required to prepare the samples, but the use of additional chemicals still remains to perform the gold reduction. Some other methodologies have shown to be quite promising. Some examples are the pre-functionalization of textile materials with cross-linking agents and plasma treatments, able to optimize the nanoparticles’ adhesion and the fabrics surface reactivity. Other studies involving silk demonstrated to be able, through the functional groups existing on the fibers, to act as reducing agents using thermal optimization (85 °C). These studies open new possibilities to research projects that include other polymeric materials to obtain in situ AuNPs synthesis without the need of additional chemicals. In addition, a few reports exist in the textiles field, combining other antimicrobial agents with AuNPs. This approach may be interesting once recent studies have displayed the improvement of the antimicrobial effect of agents already on the market, even those in which microbial resistance has been developed, in presence of AuNPs. The use of biological methods for the synthesis of AuNPs have been studied and widely applied in the two-step approach to functionalize textiles, but there are still many incompatibilities with the development of nanofibers by electrospinning and a few studies were found. The research of antimicrobial properties of these materials is still restricted to small groups of bacteria. In this sense, studying the effect of textiles developed on other bacteria strains and other microorganisms can be of great interest. In this way, several research works can be developed. The incorporation of AuNPs in textiles has shown utility in several applications, mainly in the biomedical field, given the biocompatibility of gold compared to other metals. Its use may increase with the resolution of the referred problems, particularly the use of toxic chemical reagents during synthesis. Additionally, a few studies exist about the gold release profile and cytotoxicity in these composites, however, more research should be performed. After the process optimization, textiles functionalized with AuNPs may be amplified to personal protective equipment and sports clothing.

## Figures and Tables

**Table 1:** Studies about the functionalization of fabrics (F), knitted fabrics (KF), and non-woven fabrics (NW) with AuNPs. (n.a. = not available).

Method for Synthesis of AuNPs	Deposition Method	Fabric/Textile	Precursor Salt	Reducing Agent	Stabilizing Agent	Additional Information	Size of NPs	Application	Reference
Chemical reduction	Drop-wise deposition	Cotton, silk, wool, polyester and nylon—F	Chloroauric acid (0.001 M)	Sodium borohydride solution of (0.1 M, 3 mL)	Sodium citrate 3 mL solution of 0.001 M	Sodium citrate also act as capping agent	n.a.	Wearable sensors	[48]
Chemical reduction	Padding	Cotton—F	Chloroauric acid (0.01 Wt %, 50 mL)	Trisodium citrate (1 wt%)	No stabilizing agent	Keratin coating (360 mL of keratin solution (10 mg/mL concentration).	71.8 nm	Antimicrobial textiles	[49]
Chemical reduction	Printing and paste method	Polyester—F	Gold (III) chloride hydrate	Sodium citrate	No stabilizing agent	One step green procedure	13–20 nm	Coloration, UV protection	[42]
Chemical reduction, seed-mediated growth	Immersion	Silk and cotton—F	Tetrachloroauric acid (0.01 M, 0.25 mL) Tetrachloroauric acid (0.01 M, 20 mL)	Sodium borohydride (0.01 M, 0.6 mL) Ascorbic acid (0.1 M, 3.2 mL)	CTAB (0.1 M) 9.75 mL CTAB (0.1 M) 400 mL	Au nanorods with spherical shape.	19 nm	Textile’s collation, UV protection, and antibacterial	[50]
In situ chemical reduction	Immersion and heating	Nylon—F	Tetrachloroauric (III) acid (0.05, 0.10, 0.15 and 0.20 mM)	Trisodium citrate	Trisodium citrate	pH value is in the range of 5.0–6.5 as per the concentration.	n.a.	UV blocking textiles	[51]
Chemical reduction	Exhaustion	Soybean—KF	Tetrachloroauric acid 0.01% W/V	Sodium citrate dihydrate (1% W/V, 2 mL)	Chitosan	Treatment with chitosan; spherical shape.	34.6 ± 0.5 nm	UV blocking and antimicrobial textiles	[52]
In situ chemical reduction	Impregnation	Cotton, silk, and wool—F	Hydrogen tetrachloroaurate (III) trihydrate	Sodium borohydride (1.3 g/L)	n.a.	AuNPs are mixed with other nanoparticles such as Ag and Pt; AuNPs have spherical shape.	6.64 nm	Antimicrobial textiles	[53]
In situ synthesis, groups on silk	Immersion and constant shaking at 85 °C	Silk—F	Gold (III) chloride (0.5–2 mM)	Silk macro molecular chains	Silk fabric	Hydrogen peroxide used for activation of silk macro molecules.	22–66 and 18–49 nm	Fabric coloration and antimicrobial properties	[54]
In situ synthesis, sericin from silk	Soaking and sonication	Silk—F	Tetrachloroauric (III) acid (10 mg/mL, 500 mL)	Reduction by sericin from silk	n.a.	Spherical and ellipsoidal; pH = 12.	11 ± 4 nm	Antimicrobial textiles	[55]
Chemical reduction	Immersion	Polypropylene—NW	Tetrachloroauric Acid (1 mM)	Gallic acid (0.5 mM)	Without stabilizer	Surface activation by dielectric barrier discharge (DBD) and diffuse coplanar surface barrier discharge (DCSBD).	20 nm	Antimicrobial textiles	[56]
n.a.	Deposition-precipitation	Poly(ethylene terephthalate—NW	Tetrachloroauric acid (5 mmol/L)	n.a.	n.a.	Fabric coated with ZrO2 fine particles before deposition of AuNPs at pH = 7	n.a.	Air filter	[57]
In situ chemical reduction	Immersion	Cotton—F	Tetrachloroauric acid (10 mM)	Polydopamine (2 mg/mL)	n.a.	Treated with polydopamine before depositing nanoparticles; AgNPs were deposited prior to AuNPs at pH = 8.5.	n.a.	Catalysis	[58]
In situ chemical reduction	Immersion and stirring	Cotton—F	Hydrogen tetra-chloroaurate (III) trihydrate HAuCl_4_ (1 mL, 20 mM)	N-vinyl pyrrolidone (0.1 mL)	1-Hexadecylamine	Surface modification by ATS is crucial for the formation of gold nano particles; spherical shaped.	2–7 nm	Textile coloration	[59]
Chemical reduction	Dip coating	Cotton—F	Sodium tetrachlorocuprate (III) dihydrate (1%, 90 µL)	Trisodium citrate (1%, 2.7 mL)	n.a.	Cotton fabric precoated with Zn nanorods before deposition of AuNPs.	18.5 ± 2.8 nm	Photocatalysis	[60]
In situ chemical reduction	Soaked in solution	Polyethylene-coated polypropylene—NW	Chloroauric acid	Amine groups grafted in textile surface	Amine groups grafted in textile surface	PE-coated PP fabric was used as a ligand and template; fabric is treated by the electron beam; spherical shape.	5–20 nm	n.a.	[61]
Chemical reduction	Soaked in solution of AuNPs solution	Nylon-6—F	Tetrachloroauric acid (0.0863 g)	Oleylamine	n.a.	–	14.6 ± 1.4 nm	Catalytic systems	[62]
In situ synthesis, tyrosine groups on silk fiber	Immersed in solution and heating	Silk—F	Tetrachloroauric (III) acid Various concentrations	Tyrosine groups on silk fiber	Tyrosine groups on silk fiber	Different shapes were observed according to the Wt % of the precursor solution.	21.3 ± 3.4 nm	Textile colorations and antimicrobial effect	[63]
In situ synthesis	Immersion and heating	Silk—F	Tetrachloroauric acid (0.1–0.6 mM, 50 mL)	n.a.	n.a.	Spherical, triangular nanoplates, truncated nanoprisms, and polygonal; depend on concentration of the precursor solution.	n.a.	Fabric coloration	[64]
In situ synthesis, sericin from silk	Immersion and heating	Silk—F	Tetrachloroauric (III) acid (0.3 mM)	n.a.	n.a.	The pH value of solutions was adjusted to 3; spherical and platelike shape.	n.a.	Fabric coloration and antimicrobial properties	[65]
Biological reduction	Pad-dry-cure	Cotton—F	Chloroauric acid (0.001 M, 2.5 mL)	*Acorus calamus* rhizome extract (2.5 mL)	Plant extract	Small spherical ball and bigger spherical ball, and it depends on the concentration at pH = 4, 7 and 9.2.	(0.001 M) below 100 nm (0.01 M) 100–500 nm	Antibacterial and UV blocking	[66]
Biological reduction	Sonication	Cotton and viscose—KF	Tetrachloroauric acid (3 mM, 100 mL)	Bacterial isolates (Streptomyces Sp)	Streptomyces Sp	Plasma treatment along with the combination of TiO2NPs and ZnONPs spherical shape.	4–13 nm	Antimicrobial and UV- blocking textiles	[67]
In situ synthesis, hydroxyl groups on cellulose	In situ synthesis	Cotton—KF	Tetrachloroauric acid (0.025, 0.05, 0.075, 0.10, and 0.125 mM)	Hydroxyl groups from cellulose	n.a.	Cotton also acted as a reducing agent; spherical and triangular nanoplates.	8.7 ± 1.2, 8.6 ± 1.3, 14.1 ± 3.0, 17.4 ± 3.0, and 20.5 ± 3.8 nm	Catalytic, UV blocking, and antibacterial textiles	[68]
In situ synthesis, hydroxyl groups on cellulose	Dip and dry	Cotton and polyester—F	Tetrachloroauric (III) acid (5.88 × 10^−4^ M, 198 mL,)	Hydroxyl groups on cellulose	Natural rubber latex	–	31 nm	Catalytic textiles	[69]
Biological reduction	Pad dry cure	Cotton—F	Tetrachloroauric acid (0.001 M and 0.1 M, 2.5 mL)	*Coleus aromaticus* leaf extract (2.5 mL)	*Coleus aromaticus* leaf extract	Spherical, rod, and triangular shapes, pH = 7.	Different sizes (<20 nm)	Antimicrobial textiles	[70]
Biological reduction	Pad-dry-cure	Cotton—F	Chloroauric acid (0.001 M)	*Croton sparsiflorus* leaves Extract	*Croton Sparsiflorus* leaves extract	Low concentration: bulbous shape high concentration: spherical shape; the fabric was pre-treated through scouring and bleaching.	12.2–12.7 High concertation 16.6 nm.	UV protection, antibacterial, and anticancer	[71]
Biological reduction	Dry-jet wet spinning process	Cellulose—NW	Tetrachloroauric acid (0.03 mL/g, 50 mM)	Bleached birch pre-hydrolyzed kraft pulp	n.a.	–	n.a.	UV blocking	[72]
In situ biological reduction	Immersion	Silk and cotton—F	Tetrachloroauric acid (2.00 × 10^−4^ M, 80 mL)	Ginkgo biloba Linn leaf powder extract	n.a.	Rectangular, spherical, hexagonal with smooth edges, or roughly circular in shape.	10–75 nm	Textile colorations and antimicrobial effect	[73]
In situ chemical reduction	Immersion	Silk and cotton—F	Tetrachloroauric acid (2.00 × 10^−4^ M, 80 mL)	Potassium borohydride	n.a.	–	n.a.		
In situ biological and chemical reduction	Immersion	Silk and cotton—F	Tetrachloroauric acid (2.00 × 10^−4^ M, 80 mL)	Ginkgo biloba Linn leaf powder extract and potassium borohydride	n.a.	–	n.a.		
In situ photoreduction	Immersion	Silk—F	Tetrachloroauric acid (0.2, 0.3, 0.4, 0.5, 0.6, and 0.7 mM)	n.a.	n.a.	In situ synthesized AuNPs on through the induction of sunlight; spherical shape.	16.9 ± 1.2, 24.1 ± 1.7, 23.0 ± 2.1, 20.6 ± 1.2, 19.9 ± 1.3, and 28.4 ± 1.6 nm	Fabric coloration	[74]
Heating and photochemical	Exhaustion	Wool—F	Tetrachloroauric acid (0.2 mM)	Trisodium citrate, D-Malic acid disodium salt or disodium tartrate (1 mM)	Trisodium citrate	Spherical and egg shapes were observed for heating method and photochemical synthesis at pH 4, respectively.	Various sizes	Textile coloration	[75]
n.a.	Electrodeposition	Polyester—F	Gold (III) chloride trihydrate (20 mM)	n.a.	n.a.	Coating single-walled carbon nanotubes on the polyester textile substrate before AuNPs deposition.	50 nm	Fuel cells—conductive fabrics	[76]

**Table 2:** Studies about the functionalization of Fibers (Fb), Yarns (Y) or Threads (T). (n.a. = not available).

Method for Synthesis of AuNPs	Deposition Method	Fabric/Textile	Precursor Salt	Reducing Agent	Stabilizing Agent	Additional Information	Size of NPs	Application	Reference
Photochemical reduction	Exhaustion	Silk and nylon—Fb	Tetrachloroauric acid (0.2 mM)	Trisodium citrate, D-malic acid disodium slat, and disodium tartrate (1 mM)	Trisodium citrate	Spherical and egg shapes were observed for heating method and photo chemical synthesis at pH = 4, respectively	Various sizes	Textile coloration	[75]
Chemical reduction	Soaked in solution	Cotton—T	Hydrogen tetrachloroaurate (0.65 mM)	Sodium citrate tribasic dihydrate	n.a.	SERS technique was used to detect and analyze adsorbed gold nanoparticles.	20 and 60 nm	Diagnostics for surface-enhanced Raman scattering (SERS) spectroscopy	[77]
Chemical reduction	Immersion and capillary action	Cotton—T	Tetrachloroauric acid solution 2% (V/V)	Sodium citrate (2% (M/V))	n.a.	Spherical shape; used HCl and NaOH for pH.	20–40 nm	Surface-enhanced Raman scattering detection	[78]
Chemical reduction	Sonication	Cellulose—Fb	Tetrachloroauric (III) acid (0.5 mM, 20 mL)	Sodium squarate in water	Sodium squarate in water	AuNPs synthesized in water; Spherical in shape.	21.01 nm	Catalysis	[79]
Chemical reduction	Immersion and stirring	Cotton—Y	Tetrachloroauric (III) acid (1 mM)	Trisodium citrate dihydrate (4 mM)	Citrate	pH 3–4.	13 nm	Human motion sensor/wearable sensor	[80]
In situ chemical reduction	Soaking in solution	Silk fibroin—Fb	Tetrachloroauric (III) acid (10 mmol L^−1^)	Sulfonated polyaniline (20 mL of 5 wt %)	n.a.	Sulfonated polyaniline modified fibers catalytic reduction reaction of p-nitrophenol by NaBH_4_	50–100 nm	Catalysis	[81]
In situ chemical reduction	Immersion	Ramie—Fb	Tetrachloroauric (III) acid with different concentrations	Sodium borohydride	n.a.	AuNPs were synthesized in acidic condition, pH = 2–6.	n.a.	Textile coloration and antimicrobial textiles	[82]
In situ chemical reduction	Immersion and stirring	Cellulose—Fb	Tetracholoroaurate (0.5 mM)	Sodium rhodizonate	Sodium rhodizonate	Size depends on temperature; spherical shape.	11 nm at 23 °C and 7 nm at 80 °C	Catalysis	[83]
Chemical reduction	Immersion and stirring	Cellulose—Fb	Gold chloride (AuCl_3_)	p-nitro-aniline (2 mM) Sodium borohydride (150 mM)	Cellulosic macromolecules	n.a.	26.1 nm	Catalysis	[84]
Chemical reduction	Electroless deposition	Gold/graphene—Y	Tetrachloroauric (III) acid (1.6 mM)	Hydroxylamine	n.a.	Spherical to plate; dependent on reaction time.	40 nm	Wearable electronics	[85]
Chemical reduction	Immersion	Regenerated cellulose—Fb	Gold (III) chloride triydrate (1 mM)	Trisodium citrate (1%, 2.2 mL)	Trisodium citrate	Fibers were grafted with positive charge; spherical shape	40–50 nm	colorimetry and surface-enhanced Raman scattering (SERS) assays	[86]
Chemical reduction	Sonication	Cotton—T	Tetrachloroauric (III) acid (0.01%, W/V)	Trisodium citrate	n.a.	AuNPs coated on CNTs CNTs were functionalized with PDDA; homogenous surface.	15 nm	Immunological chromatographic sensor	[87]
Chemical reduction	Centrifugation	Cotton—T	Tetrachloroauric (III) acid (0.01%, W/V)	Trisodium citrate	n.a.	AuNPs coated on CNTs CNTs were functionalized with PDDA; homogenous surface.	15 ± 3 nm	Immunological chromatographic sensor	[88]
In situ green synthesis	Immersion	Cotton—Fb	Hydrogen tetrachloroaurate (III) hydrate (0.05 mM)	*Osmanthus fragrans* 10% (m/v)	*Osmanthus fragrans*	Spherical and hexagonal shape.	40 and 60 nm	Heterogeneous catalyst	[89]
Biological reduction	Soaking	Silk—Fb	Tetrachloroauric acid (10^−3^ M)	*Citrus paradisi* extract	n.a.	Quasi-spherical	30 nm	Textile coloration	[90]

**Table 3:** Studies about the functionalization of Nanofibers (NF), Scaffolds (S) and Membranes (M). (n.a. = not available).

Deposition Method	Fabric/Textile	Precursor Salt	Reducing Agent	Stabilizing Agent	Additional Information	Size of NPs	Application	Reference
**Method for Synthesis: Biological reduction**								
Electrospinning solution	Polyacrylonitrile—NF	Tetrachloroauric (III) acid (0.001 M)	Banana peel extract, phenolic compounds and flavonoids	n.a.	Spherical shape.	9 nm	Electrochemical sensors	[2]
**Method for Synthesis: Chemical reduction**								
Electrospinning solution	Polyimide—NF	Gold (III) chloride hydrate	Thermal treatment at 200 °C in polyimide nanofibers	n.a.	Thermally reduced Au3+ to Au0.	n.a.	n.a.	[18]
Immersion	Crystalline cellulose—NF	Tetrachloroauric acid	Sodium borohydride	n.a.	-	<5 nm	Catalysis	[91]
Immersion	Bacterial cellulose—NF	Tetrachloroauric acid	Poly (ethyleneimine)	Poly(ethyleneimine)	Spherical shape.	≈9 nm	Biosensors	[92]
Immersion	Bacterial cellulose—NF	Tetrachloroauric acid (20 mM)	Poly (ethyleneimine)	n.a.	BC nanofibers are produced by ultrasonic cell disruption system at room temperature.	≈9 nm	Biosensors	[93]
Immersion	Cellulose acetate (mats)—NF	Tetrachloroauric (III) acid (1 Wt %, 3 mL)	Trisodium citrate	Trisodium citrate	LBS self-assembly technique.	–	Antimicrobial textiles	[94]
Immersion	Thermoplastic polyurethane—NF	Hydrogen tetrachloroaurate	Trisodium citrate	Chitosan	Precoated with chitosan reduction in 4-nitrophenol by sodium borohydride, pH value 3–11.	16 nm	Catalysis	[95]
Electrospinning solution	PCL/Gelatin—NF/S	Tetrachloroauric (III) acid	Sodium borohydride	n.a.	Process is done in the presence of antibiotic intermediates.	3 nm	Antimicrobial textiles, wound treatment	[96]
Soaking	Bacterial cellulose—M	Tetrachloroauric (III) acid (40 mg mL^−1^)	Sodium borohydride	n.a.	Gold nanoparticles modified with 4,6-Diamino-2-Pyrimidinethiol (DAPT).	≈3 nm	Antimicrobial textiles, wound treatment	[97]
Electrospinning solution	PEI/PVA—NF	Chloroauric acid (0.5 mM)	Sodium borohydride	n.a.	The fibrous were cross-linked via glutaraldehyde (GA) vapor to produce water-stable fibrous mats; round shaped.	11.8 nm	Catalysis	[98]
Electrospinning solution	Polycaprolactone—NF	Chloroauric acid (0.5 mM)	Sodium borohydride (1.25 mM)	n.a.	Round shape.	5–6 nm	Heterogeneous catalysis and SERS	[99]
Immersion and shaking	PET track-etched micro-porous—M	Tetrachloroauric (III) acid (1 mg mL^−1^)	Dopamine on membranes	n.a.	Coated with dopamine.	n.a.	Catalysis	[100]
Dropping	Polyamide—NF/M	Hydrogen tetrachloroaurate (III) (1.00 mM)	Trisodium citrate dihydrate (0.30 M)	Trisodium citrate dihydrate	Spherical	20.1 ± 1.76 nm	Colorimetric sensor	[101]
Immersion	Bacterial cellulose—NF	Chloroauric acid (0.4 mM)	Sodium borohydride (6 mM)	n.a.	2,2,3,3-tetramethylpiperidine-l-oxyl (TEMPO)-oxidized (TOBCNS); spherical shape.	4.30 ± 0.97 nm	Catalysis	[102]
Immersion	Cellulose—NF	Tetrachloroauric (III) acid solution (0.2 mM)	Hydrazine hydrate	n.a.	Unsupported AuNPs.	18.3 ± 3.5 nm	Catalysis	[103]
**Method for Synthesis:** In situ **chemical reduction**								
Electrospinning solution	Polyacrylonitrile—NF	Hydrogen tetrachloroaurate (III) trihydrate	4-(Dimethylamino) benzaldehyde	n.a.	–	6 nm	Biosensors	[104]
Soaking	Cellulose Acetate—M	Tetrachloroauric (III) acid	Dithiothreitol (DTT)	Porous fiber network	DTT capped AuNPs.	2.5 ± 0.5 nm	Sensors	[105]
Electrospinning solution	Zein—NF	Tetrachloroauric (III) acid (20 mM, 0.25 mL)	Poly(ethyleneimine)	n.a.	Spherical shape; value of pH 3.0–7.0.	90.9 nm	Biosensors	[106]
Electrospinning solution	Polyacrylonitrile—NF	Tetrachloroauric (III) acid (0.30 mmol)	n.a.	n.a.	Au-PANF was prepared and electrospun to form mats; spherical shape.	2.3 ± 0.5 nm	Sensors	[107]
Electrospinning solution	Polyimide—NF	Tetrachloroauric (III) acid (0.5, 1, and 3 Wt %)	Polyimide and high temperature (200 °C)	Polyimide	Reduction takes place due to thermal reactions.	9–22 nm	High temperature end-of-service indicators	[108]
Immersion	Cellulose—M	Potassium gold (III) chloride	Sodium borohydride	Poly(diallyl-dimethylammonium chloride) poly(sodium-p-styrenesulfonate)	Membranes previously coated with titania gel.	3.5 ± 0.7 nm	n.a.	[109]
Immersion and continuous shaking	PET track-etched microporous—M	Tetrachloroauric (III) acid (1 mg mL^−1^)	Sodium borohydride	n.a.	Coated with dopamine.	n.a.	Catalysis	[100]
**Method for Synthesis: Photoreduction**								
Electrospinning solution	Polystyrene (mats)—NF	Tetrachloroauric (III) acid (3% (W/W)	Ultraviolet irradiation	n.a.	Undergone electrospinning and then mats exposed to UV light.	n.a.	n.a.	[110]
**Method for Synthesis:** In situ **photoreduction**								
Electrospinning solution	Polyacrylonitrile—NF	Gold (III) chloride hydrate(0.044 M, 0.022 M)	Sodium alginate	n.a.	UV light has been used during electrospinning; spherical shape.	21.4 nm (0.044 M) 5.8 nm (0.022 M)	n.a.	[111]
Electrospinning solution	Polyacrylonitrile—NF	Tetrachloroauric (III) acid	N,N-dimethylformamide	n.a.	Undergone electrospinning and then mats exposed to UV light.	4.7–5.4 nm (5 days of UV radiation)	n.a.	[112]
Immersion	Cellulose—NF	Tetrachloroauric (III) acid solution (0.2 mM, 20 mL)	Cellulose nanocrystals	Cellulose nanocrystals	CNs were produced from microcrystalline cellulose.	30.5 ± 13.4 nm	Catalysis	[103]
Immersion	Bacterial cellulose—M	Tetrachloroauric acid (0.2, 0.4, 0.6 mM)	n.a.	n.a.	Xenon lamp was used in the process of synthesis of AuNPs; spherical shape.	n.a.	Sensors	[113]
**Method for Synthesis: Laser ablation**								
Electrospinning solution	Polyacrylonitrile—NF	Gold plate	n.a.	n.a.	Spherical shape; face centered cubic crystal structure with crystallite size of 8 nm.	17 nm	Glucose sensors	[114]

**Table 4:** Antibacterial results of AuNPs functionalized fabrics. (n.a. = not available).

Fabric	Test Standards	Test Method	Bacterial Used	Results (Synthesis/Antimicrobial)	Reference
Cotton—F	n.a.	Kirby–Bauer	*K. pneumoniae P. aeruginosa, S. aureus,* and *E. coli*	AuNPs-keratin, 71.8 nm, sodium citrate/ Zone diameters ranging from 3.5 to 3.6 cm.	[49]
Polyester—F Cotton—F	AATCC 147-1988	Parallel streak method	*S. aureus, B. cereus, E. coli,* and *C. utilis*	AuNPs, 13–20 nm, sodium citrate/ Zone diameters ranging from 16 to 22 mm Polyester and cotton fabrics printed with AuNPs showed excellent antimicrobial activity in all tested strains.	[42]
Soybean—KF	ASTM-E2149-01	Shake flask method	*S. aureus* and *E. coli*	AuNPs-chitosan, 34.6 ± 0.5 nm, sodium citrate/ 99.94% against *S. aureus* (3.23 of log reduction) and 96.26% (1.43 of log reduction) against *E. coli.*	[52]
Cotton, silk, and wool—F	n.a.	Shake flask method	*E. coli* (ATCC 8099), *S. aureus* (ATCC 6538)	(HBPAA)-encapsulated Au-Ag-PtNPs, 6.64 nm/ Coating of nanoparticles have improved the antibacterial efficacy, and it enhanced linearly with the concentration.	[53]
Silk—F	n.a.	Turbidimetric method	*S. aureus* (ATCC 47077) and *E. coli* (ATCC 25922)	AuNPs, 22–66 and 18–49 nm, hydrogen peroxide/ Optical density reduced from 2.5 to 1.11–1.23 for *E. coli* and 2.12 to 0.93–1.11 for *S. aureus* after 24 h incubation.	[54]
Silk—F	Broth microdilution method	Broth microdilution method, inhibition zone	*S. aureus* and *E. coli*	AuNPs-gentamicin, 11±4nm/ Inhibition zone against *E. Coli* 25.8 mm, *S. aureus* 26 mm.	[55]
Polypropylene—F	n.a.	Agar diffusion test	*S. aureus* (ATCC 6538) *E. coli* (ATCC 11229)	AuNPs, 20 nm, gallic acid/ Activity only in contact; *S. aureus* was more sensitive, and the best antibacterial activity was shown for PP nonwoven fabrics treated with DCSBD (12 s (24 J/cm^2^) and DBD 120 s (14.4 J/cm^2^).	[56]
Silk—F	AATCC 100-2004 (Clause 10.2) test standard with slight modification	Agar plate method	*E. coli* (ATCC 11229)	AuNPs, 21.3 ± 3.4 nm, tyrosine on silk/ No bacteria colonies found on agar medium of silk fabrics treated with gold NPs; AuNPs on silk inhibited growth of bacteria.	[63]
Silk—F	AATCC 100-2012 (Clause 10.2) standard with slight modifications	Shake flask method	*E. coli* (ATCC 25922)	AuNPs, no size determination/ CFU reduction values of 99.96%.	[65]
Cotton—F	AATCC 100	Agar plate method	*S. aureus* (MTCC 96) and *E. coli* (MTCC1671)	AuNPs, 10 nm, A. calamus rhizome extract/ Percentage reduction 50.05% and 63.9% against *S. aureus* after 24 and 48 h culture, respectively; 58.0% and 80.3% against *E. coli* after 24 and 48 h culture, respectively.	[66]
Cotton and viscose—KF	n.a.	Agar plate method and expressed as zone inhibition method	*S. aureus* and *E. coli*	AuNPs-ZnONPs, 4–13 nm, *Streptomyces sp.* isolates/ Plasma-treated AuNPs/ZnONPs showed the highest values. For cotton inhibition zone against *E. Coli* was 27.5 mm, and for *S. aureus* was 30 mm. For viscose inhibition zone against *E. Coli* was 25 mm, and against *S. aureus* was 27 mm.	[67]
Cotton—KF	AATCC 100-2004 (clause 10.2) test standard with slight modifications	Agar plate method	*E. coli* (ATCC 11229)	AuNPs, 8.7–20.5 nm, cotton hydroxyl groups/ No bacteria colonies were found on the agar medium of the AuNP-treated cotton fabric; inhibit the growth of bacteria.	[68]
Cotton—F	n.a.	Disc diffusion susceptibility assay using MHA medium, inhibition zone	*S. epidermidis* (ATCC 35984) and *E. coli* (ATCC 10546)	AuNPs, <20 nm, *Coleus aromaticus* leaf extract/ Inhibition zone against *E. Coli* was 27 mm and *S. Epidermidis* was 22 mm.	[70]
Cotton—F	n.a.	Disc diffusion susceptibility assay, inhibition zone	*E. coli* (MTCC 10546) and *S. epidermidis* (MTCC 35984)	AuNPs, 12.2–12.7, *Croton sparsiflorus* leaf extract/ 30 and 26 mm diameter against *S. epidermidis* and *E. coli*, respectively.	[71]

**Table 5:** Antibacterial results of AuNPs functionalized electrospun fibers. (n.a. = not available).

Fabric	Test Standards	Test Method	Bacterial Used	Results (Synthesis/Antimicrobial)	Reference
Cellulose acetate electrospun mats	n.a.	Disc diffusion susceptibility assay, inhibition zone	*S. aureus* and *E. coli*	AuNPs-lysozyme, 18.7 nm, sodium citrate/ Composite mats have revealed significant antibacterial activity. The inhibition activity was better against *S. aureus* than *E. coli*.	[94]
Bacterial cellulose membrane	n.a.	Shake flask test	*E. coli (ATCC1175), P. aeruginosa* (1.2387), MDR *E. coli* (Q1124)*,* and MDR *P. aeruginosa* (N3966)	AuNPs-DAPT, 3 nm, sodium borohydride/ Au-DAPT NPs inhibited *E. coli* and MDR *E. coli* at a MIC of 4 µg/mL and inhibited *P. aeruginosa* and MDR *P. aeruginosa* at MIC of 8 and 16 µg/mL, respectively. Additionally, it promotes wound repair.	[97]

## Data Availability

Not applicable.

## Abbreviations

American Association of Textile Chemists and Colorists (AATCC), Gold Chloride (AuCl_3_), Tetracholoroaurate Ion (AuCl^4−^), Gold Nanoparticles (AuNPs), American Society for Testing and Materials (ASTM), American Type Culture Collection (ATCC), Adenosine Triphosphate (ATP), (3-aminopropyl) Triethoxy Silane (ATS), Bacterial Cellulose (BC), Cationic Polyacrylamide (CPAM), Carcinoembryonic antigen (CEA), Colony Forming Units (CFU), Cellulose Single Nanofibers (CSNFs), Hexadecyltrimethylammonium Bromide (CTAB), 4,6-Diamino-2-Pyrimidinethiol (DAPT), Dielectric Barrier Discharge (DBD), Diffuse Coplanar Surface Barrier Discharge (DCSBD), Gentamicin Sulfate (GM), Tetrachloroauric Acid (HAuCl_4_), Hydrochloric Acid (HCl), Potassium Borohydride (KBH_4_), Nanoparticles (NPs), Sodium Borohydride (NaBH_4_), Natural Rubber Particles (NRP), Natural Rubber Latex (NRL), Polyacrylonitrile (PAN), Polycaprolactone (PCL), Polydopamine (PDA), Poly(diallyldimethylammonium chloride) (PDDA), Polyethylene Glycol (PEG), Polyethyleneimine (PEI), Polyethylene Terephthalate (PET), Polyethylene (PE), Polypropylene (PP), N-Vinyl Pyrrolidone (PVP), Multi Drug Resistant (MDR), Microbial Type Culture Collection (MTCC), Oleyl Amine (OAm), 2,3,3-tetramethylpiperidine-l-oxyl (TEMPO)-oxidized (TOBCNS), Titanium Oxide Nanoparticles (TiO_2_NPs), Trioctyl-Phosphine (TOPO), Zirconium Dioxide (ZrO_2_), Zinc Oxide (ZnO).
